# Elevated dietary linoleic acid increases gastric carcinoma cell invasion and metastasis in mice

**DOI:** 10.1038/sj.bjc.6605881

**Published:** 2010-09-14

**Authors:** T Matsuoka, J E Adair, F B Lih, L C Hsi, M Rubino, T E Eling, K B Tomer, M Yashiro, K Hirakawa, K Olden, J D Roberts

**Affiliations:** 1 The Laboratory of Molecular Carcinogenesis, National Institute of Environmental Health Science, NIH, Research Triangle Park, NC 27709, USA; 2 Department of Surgical Oncology, Osaka City University Graduate School of Medicine, 1-4-3 Asahi-machi, Abeno-ku, Osaka 545-8585, Japan; 3 The Laboratory of Structural Biology, National Institute of Environmental Health Science, NIH, Research Triangle Park, NC 27709, USA

**Keywords:** gastric carcinoma, dietary fatty acid, cyclooxygenase, metastasis, invasion

## Abstract

**Background:**

Dietary (n-6)-polyunsaturated fatty acids influence cancer development, but the mechanisms have not been well characterised in gastric carcinoma.

**Methods:**

We used two *in vivo* models to investigate the effects of these common dietary components on tumour metastasis. In a model of experimental metastasis, immunocompromised mice were fed diets containing linoleic acid (LA) at 2% (LLA), 8% (HLA) or 12% (VHLA) by weight and inoculated intraperitoneally (i.p.) with human gastric carcinoma cells (OCUM-2MD3). To model spontaneous metastasis, OCUM-2MD3 tumours were grafted onto the stomach walls of mice fed with the different diets. In *in vitro* assays, we investigated invasion and ERK phosphorylation of OCUM-2MD3 cells in the presence or absence of LA. Finally, we tested whether a cyclooxygenase (COX) inhibitor, indomethacin, could block peritoneal metastasis *in vivo*.

**Results:**

Both the HLA and VHLA groups showed increased incidence of tumour nodules (LA: 53% HLA: 89% VHLA: 100% *P*<0.03); the VHLA group also displayed increased numbers of tumour nodules and higher total volume relative to LLA group in experimental metastasis model. Both liver invasion (78%) and metastasis to the peritoneal cavity (67%) were more frequent in VHLA group compared with the LLA group (22% and 11%, respectively; *P*<0.03) in spontaneous metastasis model. We also found that the invasive ability of these cells is greatly enhanced when exposed to LA *in vitro*. Linoleic acid also increased invasion of other scirrhous gastric carcinoma cells, OCUM-12, NUGC3 and MKN-45. Linoleic acid effect on OCUM-2MD3 cells seems to be dependent on phosphorylation of ERK. The data suggest that invasion and phosphorylation of ERK were dependent on COX. Indomethacin decreased the number of tumours and total tumour volume in both LLA and VHLA groups. Finally, COX-1, which is known to be an important enzyme in the generation of bioactive metabolites from dietary fatty acids, appears to be responsible for the increased metastatic behaviour of OCUM-2MD3 cells in the mouse model.

**Conclusion:**

Dietary LA stimulates invasion and peritoneal metastasis of gastric carcinoma cells through COX-catalysed metabolism and activation of ERK, steps that compose pathway potentially amenable to therapeutic intervention.

Dietary variables, including dietary fats, total energy and micronutrients clearly influence carcinogenesis in animal models, (for review, see Sauer *et al*, 2007). Epidemiological evidence suggests that in some cases, these findings are relevant to human cancers (Michels *et al*, 2007). Dietary variables may explain the changing frequency of cancer seen in immigrants, who acquire the dietary habits of their adopted country (Armstrong and Doll, 1975). However, controlled prospective studies have not always demonstrated major effects of dietary fat on cancer risk (for example, see Prentice *et al*, 2007). Experimental evidence relating dietary fat and tumour development has been obtained from animal models for a variety of cancers (De Vries and van Noorden, 1992; Connolly *et al*, 1996; Fischer *et al*, 1996; Rose, 1997b; Rose and Connolly, 1998), including some gastric carcinomas (Silva *et al*, 1995; Lopez-Carrillo *et al*, 1999). Specifically, (n-6)-polyunsaturated fatty acids (PUFAs) have been shown to promote carcinogenesis (Braden and Carroll, 1986) and stimulate metastatic behaviour of tumour cells (Hubbard and Erickson, 1987; Rose, 1997b). Linoleic acid (LA), an important (n-6)-PUFA, is metabolised in a variety of ways, including conversion to arachidonic acid (AA) (Lichtenstein *et al*, 1998), a precursor of a variety of highly active eicosanoids formed through cyclooxygenase (COX)- and lipoxygenase (LOX)-mediated reactions (Zha *et al*, 2004; Furstenberger *et al*, 2006). The COX-1 is constitutively expressed in most tissues, generating basal levels of prostaglandins, whereas COX-2 is generally expressed at lower levels and can be induced by a variety of extracellular stimuli (Kujubu *et al*, 1991). One epidemiological study showed that the risk of developing stomach cancers was reduced in patients taking a COX inhibitor (Farrow *et al*, 1998), and metastasis was suppressed by non-steroidal anti-inflammatory drugs, inhibitors of these key enzymes (Vanderhoek *et al*, 1984).

Gastric carcinoma is one of the most frequent and lethal malignancies in the world (Parkin *et al*, 1999). Aggressive scirrhous gastric carcinoma, a diffusely infiltrating adenocarcinoma, is characterised by rapid progression, invasion and frequent dissemination into the abdominal cavity, leading to peritoneal metastasis (Sowa *et al*, 1989). The choice of treatment depends on knowledge of the natural history of the disease and mechanisms of spread. Because such information is generally lacking, various treatment regimens have been disappointing and advanced gastric carcinoma remains incurable, with a median survival of 6–10 months (Park *et al*, 2004). We have previously shown that PUFAs promote the metastasis of breast carcinoma cells by a mechanism involving stimulation of adhesion to collagen type IV and activation of PKC and p38 MAPK pathways (Palmantier *et al*, 1996; Paine *et al*, 2000). Whether fatty acids promote the metastasis of gastric carcinoma in a mouse model is the focus of this report.

## Materials and methods

### Cell lines and cell culture

A highly metastatic human gastric carcinoma cell line, OCUM-2MD3, was established from a parental human cell line, OCUM-2M, using orthotopic tissue implantation in nude mice (Nishimura *et al*, 1996; Yashiro *et al*, 1996; Matsuoka *et al*, 1998). Another gastric carcinoma cell line, OCUM-12, was also provided by Dr Kosei Hirakawa, Osaka City University, Osaka, Japan. NUGC3 and MKN-45 were obtained from JCRB cell bank (Osaka, Japan). Cells were maintained in Dulbecco's modified Eagle's medium (DMEM, Life Technologies, Grand Island, NY, USA), supplemented with 10% heat-inactivated fetal bovine serum (HyClone, Logan, UT, USA) (complete DMEM), at 37°C in a humidified atmosphere containing 5% CO_2_.

### Antibodies and reagents

Linoleic acid, LA, AA and nordihydroguaiaretic acid were from Cayman Chemical Co., Ann Arbor, MI, USA. Protease inhibitors were from Sigma-Aldrich, St Louis, MO, USA or Chemicon, Billerica, MA, USA. International Sources of primary antibodies were as follows: ERK1/2 and phospho-ERK1/2, Upstate Biotechnology, Lake Placid, NY, USA; phospho-JNK, New England Biolabs, Ipswich, MA, USA; actin, Santa Cruz Biotechnology, Santa Cruz, CA, USA; phospho-p38, Promega, Madison, WI, USA; COX-1, Cayman; COX-2, Oxford Biomedical, Oxford, MI, USA; and GAPDH, Chemicon International. The COX-1 and COX-2 standards for gel electrophoresis were partially purified preparations from Cayman.

### Animals and diets

Female athymic nude mice (NCr-*nu/nu*; 4-week-old) were from Charles River Laboratories Portage, MI, USA and were maintained in micro-isolator cages in a pathogen-free facility. All studies were conducted in accordance with the NIH Guide for the Care and Use of Laboratory Animals, and all experiments were approved by the NIEHS Institutional Animal Care and Use Committee. Mice were divided into three groups of 19 mice each for the experimental metastasis study (injection of tumour cells into the intraperitoneal (i.p.) space), and two groups of 10 mice each for the spontaneous metastasis study (tumour fragments sutured onto the stomach wall). The mean weight per mouse in each group was the same. Diets were based on the purified AIN-76A diet (Rose *et al*, 1993) and prepared by BioServe Inc., Frenchtown, NJ, USA. The diets were isocaloric (4.45 kcal g^−1^) and contained the same high-fat content (23%, w/w), supplemented with LA-rich safflower oil and saturated fatty acid-containing coconut oil in different ratios to yield 2% (LLA), 8% (HLA) or 12% (VHLA) (w/w) LA (Supplementary Tables 1 and 2) (Hubbard and Erickson, 1987). Diets were sterilised by irradiation with ^60^Co and stored at 4°C in heat-sealed plastic bags flushed with nitrogen. Feeding of the experimental diets commenced 7 days before injection or transplantation of the tumour cells.

### *In vivo* metastasis models

Cells were harvested from subconfluent cultures, collected by centrifugation, washed once and resuspended in serum-free DMEM at 1 × 10^7^ cells per ml. For experimental metastasis, mice were injected i.p. with 2 × 10^6^ cells in 200 *μ*l, maintained on experimental diets for an additional 4 weeks and then killed using CO_2_. The maximum *(L)* and perpendicular *(W)* diameters of metastatic nodules at necropsy were measured with a vernier caliper and the volume of nodules was calculated as follows: 
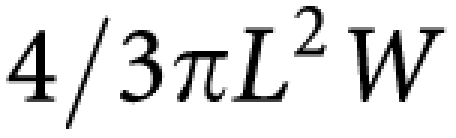


For spontaneous metastasis studies, tumour fragments for implantation were prepared by injecting nude mice subcutaneously with 5 × 10^6^ OCUM-2MD3 cells. At 4 weeks after inoculation, subcutaneous tumours were excised and cut into 3-mm square pieces. Additional nude mice, maintained on the appropriate diet, were anaesthetised and an incision made through the median upper abdominal line and peritoneum. The stomach was exposed, and a single tumour fragment was sutured onto the middle of the greater curve of the glandular stomach. At 4 weeks after implantation, mice were killed and the stomach and any metastatic colonies were removed. Visible tumours were counted, measured, fixed in 10% formalin, embedded in paraffin and cut into 2-mm-thick slices for histological examination.

Indomethacin (Sigma-Aldrich) was dissolved in absolute ethanol, diluted as required to provide 10 mg indomethacin per l (0.2% ethanol) in the drinking water (Lala *et al*, 1997) and administered starting 7 days before injection of tumour cells and throughout the study. Control mice received ethanol (0.2%) in the drinking water. Systemic toxicity was not evident in any of these mice.

### Cell growth assays

The OCUM-2MD3 cells were plated at a density of 1 × 10^4^ cells per well in 96-well plates. Cells were washed with phosphate-buffered saline (PBS), incubated with or without LA (3, 30 or 60 *μ*M) for 72 h and then treated with 5 mg ml^−1^ MTT (3-(4,5-dimethyl-2-thiazyl)-2,5-diphenyl-2*H*-tetrazolium bromide) (Sigma-Aldrich) for 4 h at 37°C. The relative active number of cells was measured as absorbance at 570 nm using a microtiter plate reader (PM2004; Wako, Osaka, Japan).

### Flow cytometry

Apoptosis was detected using flow cytometry by staining cells with annexin V-FITC and propidium iodide (BD Pharmingen, San Diego, CA, USA) labelling. The OCUM-2MD3 cells treated with vehicle (ethanol) and 30 *μ*mol l^−1^ of LA for 30 min were seeded at a density of 1.0 × 10^5^ cells per ml in 100-mm plates. Cells were stained with annexin V-FITC and propidium iodide and analysed by flow cytometry using FACScan.

### Invasion assay

Tumour cell invasion was assayed as described previously with some modifications (Albini *et al*, 1987). Briefly, Transwell cell culture chambers (Millipore Co., Billerica, MA, USA), equipped with a microporous membrane filter (pore size: 12 *μ*m), were coated with Matrigel (10 *μ*g per filter) (Becton Dickinson, Bedford, MA, USA). The OCUM-2MD3 cells, and the other scirrhous gastric carcinoma cell lines, OCUM-12, NUGC3 and MKIN-45, were resuspended to 5 × 10^5^ cells per ml in complete DMEM containing either 30 *μ*mol l^−1^ LA or the appropriate amount of vehicle (ethanol), added to the upper compartment of the chamber and incubated at 37°C in 5% CO_2_. We choose the LA concentration from previously published data, such as Rose *et al* (1994). Similarly, the effect of 30 *μ*mol l^−1^ AA, a metabolite of LA, on the invasion of OCUM-2MD3 cells was investigated. When relevant, cells were treated with the ERK inhibitor, PD98059 (Calbiochem, La Jolla, CA, USA), or p38 inhibitor, SB203580, for 1 h, or with the COX inhibitor, indomethacin, or LOX inhibitor, NDGA, for 30 min; control cells received vehicle only. Invasion was assessed by counting cells that appeared on the underside of the membrane, counting three fields (magnification, × 200) per well and three wells per experimental group.

### Preparation of cell lysates for western blotting

Subconfluent cells were washed with serum-free medium, resuspended at 5 × 10^5^ cells per ml and allowed to recover in 5% CO_2_ at 37°C for 20 min. Specific ERK and p38 inhibitors, PD98059 and SB203580 (Calbiochem), respectively, or indomethacin, were dissolved in DMSO and added to cells 1 h (PD98059 and SB203580) or 30 min (indomethacin) before treatment with 30 *μ*mol l^−1^ of LA for the indicated time period. Cells were collected by centrifugation and resuspended in RIPA buffer (50 mmol l^−1^ Tris-HCl (pH 7.5), 30 mmol l^−1^ Nonidet P-40, 150 mmol l^−1^ NaCl, 200 IU l^−1^ aprotinin, 1 mg l^−1^ leupeptin, 0.5 mmol l^−1^ sodium orthovanadate, 50 mmol l^−1^ sodium fluoride, 2 mmol l^−1^ EDTA, 1% Triton X-100, 1% sodium deoxycholate and 0.1% SDS). Cell lysates were then passed through a 23-G needle and clarified by centrifugation. Protein concentrations were determined using the BCA protein assay (Pierce, Rockford, IL, USA). With mouse tissue samples, 100–500 mg of each sample was crushed with a mortar and pestle in a liquid nitrogen bath. After the addition of five volumes of ice-cold 50 mmol l^−1^ Tris-HCL (pH 7.4), 1 mmol l^−1^ EDTA, samples were homogenised, centrifuged and supernatants used for immunoblot analysis.

### Immunoblots

Whole cell lysates were resolved by 10% SDS-PAGE and transferred to PVDF membranes (Millipore Co.) in Tris-glycine buffer containing 10% methanol. Membranes were blocked with 5% skim milk or 3% BSA (fraction V, MP Biomedicals, Solon, OH, USA) in Tris-buffered saline with 0.1% Tween 20 (TBST), incubated with primary antibodies at room temperature for 1 h or at 4°C overnight, followed by the appropriate secondary antibody at room temperature for 45 min. After incubations with primary and secondary antibody, blots were washed with TBST and visualised using either Supersignal chemiluminescent substrate (Pierce) or Amersham ECL detection system, Piscataway, NJ, USA.

### Immunohistochemical detection of the COXs

The streptavidin–biotin peroxidase complex method was used for immunohistochemistry. Slides of tissue samples were treated with 0.3% hydrogen peroxide in methanol for 45 min. Antibodies to COX-1 and COX-2 were applied to sections at a dilution of 5 and 10 mg l^−1^, respectively, with an overnight incubation at 4°C. Sections from previously studied cases of colorectal cancer known to express COX-2 were used as positive controls. Antibody binding was detected using a Vecta Stain Elite kit (Vector Laboratories, Burlingame, CA, USA) and counterstained with haematoxylin.

### RT-PCR detection of COX-1 mRNA

Quantitative reverse transcription (RT)-PCR was used to determine relative expression levels of COX-1 mRNA. Total RNA from tissues was isolated using the RNeasy Midi Kit (Qiagen, Valencia, CA, USA). A measure of 1 *μ*g of total RNA from each tissue was reverse transcribed by Superscript II (Invitrogen, Carlsbad, CA, USA). Primers chosen for the cloned 300-bp PCR fragment were: sense 5′-TGCCCAGCTCCTGGCCCGCCGCTT-3′ antisense 5′-GTGCATCAACACAGGCGCCTCTTC-3′. The PCR reactions were denatured for 4 min at 94°C, followed by 40 cycles at 94°C for 1 min, 63°C for 1 min and 72°C for 1 min. For *β*-actin amplification (sense primer 5′-CGGGGACCTGACTGACTACC-3′ antisense primer 5′-AGGAAGGCTGGAAGAGTGC-3′), reactions involved denaturation at 94°C for 1 min, annealing at 57°C for 1 min and extension at 72°C for 1 min. The PCR products were separated on 2% agarose gels and visualised using ethidium bromide.

### Fatty acid profiling by mass spectrometry

Duodenal tissues for extraction were excised, rinsed thoroughly in PBS three times and weighed (mass ranged from 29 to 57 mg). Sufficient water was added to each sample to bring the total mass of water and tissue to 60 mg. For blood plasma extractions, a 25 *μ*l volume of separated plasma was used. Subsequent volumes were reduced by 5/12 to maintain solvent ratios. All samples received 2.5 *μ*l of butylated hydroxytoluene (as an antioxidant) in methanol at a concentration of 10 g l^−1^. Lipids were then extracted from duodenal tissue and blood plasma by the Folch procedure (Folch *et al*, 1957) and 0.5 *μ*l of each sample was loaded onto the column. Fatty acid methyl ester peaks were identified by comparing spectra with those in the NIST mass spectral library, and by comparing them with spectra and retention times of Supelco 37 Component FAME Mix (Sigma-Aldrich). Relative abundances were determined from the total ion chromatogram. A correction factor was applied to convert peak areas to weight percentages on the basis of the results obtained from analysis of the Supelco FAME mix. Mean weight percentages and standard deviations were calculated for four biological replicate samples from each diet group.

### Statistical analysis

Analysis of incidence was conducted using Fisher's exact tests. Incidence data represented in Table 1 were analysed by two-sided Fisher's exact test, as the effect of LA on tumour formation in this experiment was unknown. Incidence data represented in Tables 2 and 3 were analysed by one-sided Fisher's exact tests based on experimental data from Table 1, which suggested an increase in tumour formation as a function of LA. All data are reported as mean±standard error of the means.

In the analysis of fatty acids present in duodenal tissue and plasma (Supplementary Table 3 and 4, respectively), residuals were examined to verify the shape of the distribution as normal, given the small sample size used in this analysis. On the basis of these results, parametric analysis was used to determine the significance of observed changes in fatty acids as a function of dietary LA. Data are reported as mean±standard deviation (s.d.).

To determine whether indomethacin had a significant effect on number of tumour nodules per animal and the total tumour volume per animal in combination with a low- or high-LA diet *in vivo* (Table 3), two-way analysis of variance (ANOVA) for independent samples was utilised. Data are reported as mean±s.d.

*In vitro* data comparing treated and control groups and *in vivo* data comparing number of nodules and total tumour volume per animal were analysed using the Student's *t*-test. Data are reported as means±s.d. In all cases, *P*-values of <0.05 were considered statistically significant.

## Results

### LA stimulates metastasis of a human gastric cancer cell line

**Figure 1:**
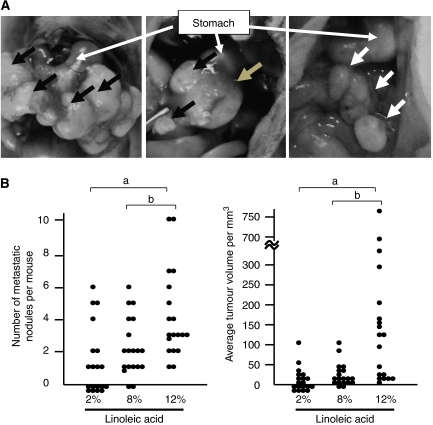
The effect of LA on the metastasis of a human gastric cancer cell line. (**A**) Typical views of the abdominal space in a euthanised mouse fed the 12% LA diet are shown. *Left panel*: metastatic lymph nodes around stomach (black arrows) in the model of orthotopic implantation. *Middle panel*: primary gastric implanted tumour (grey arrow). *Right panel*: metastatic peritoneal tumour (white arrows) in the experimental metastasis model. (**B**) Number of metastatic nodules per mouse, counted 4 weeks after i.p. injection of OCUM-2MD3 cells. Bars indicate the mean. a: Different from LLA group (*P*<0.03). b: Different from HLA group (*P*<0.03 by the Jonckheere–Terpstra test). Points at the abscissa indicate mice with no detectable tumour. Bars represent the mean for each group.

To determine whether LA would stimulate metastasis of gastric carcinoma cells, OCUM-2MD3 human gastric carcinoma cells were injected into the peritoneal cavities of Balb/c nude mice, which were fed high-fat diets containing 2% (LLA), 8% (HLA) or 12% (VHLA) LA. Mean per mouse body weights did not differ between the three dietary groups at either the beginning or the end of the study (Table 1). Both the HLA and VHLA groups showed a higher incidence of metastasis compared with the LLA group (Table 1), despite identical total fat and energy intakes (Supplementary Table 1). Interestingly, the mean number of metastatic nodules per mouse and the mean total tumour volumes in the VHLA group increased over those in either the LLA or HLA groups (Table 1 and Figure 1B). Metastatic colonies, identified as white nodules in the peritoneal cavity (Figure 1A, right panel), were primarily located at the omentum and occasionally at the lesser curvature of stomach, mesentery and parietal peritoneum. Tumour location did not vary between dietary groups.

We next used orthotopic implantation of tumours on the stomach wall to examine the effects of dietary LA on OCUM-2MD3 tumour growth and spontaneous peritoneal metastases. Because the VHLA group showed the largest effect in the earlier model, and to reduce the number of mice used in this model, in which donor and recipient mice are required, we chose to test for an effect on spontaneous metastasis in mice fed only the LLA and VHLA diets. All the mice receiving tumour cell transplants developed detectable tumours on the stomach wall (Figure 1A, middle panel). The mean volume of the original engrafted tumours in the VHLA group was not different from that of the LLA group (*P*=0.0854) (Table 2), suggesting that there were no effects of the diet on growth of the original tumour cells. Metastatic colonies, identified by histological examination, appeared both in the peritoneal cavity and in lymph nodes, but not in the lungs.

Metastasis to the peritoneum and liver invasion were significantly increased in the VHLA group (Table 2), whereas lymph node metastasis (Figure 1A, left panel) did not increase when compared with the LLA group (*P*=0.31), suggesting that there is organ specificity for the effect of dietary LA on gastric carcinoma metastasis. However, as the level of lymph node metastases was already substantial in the LLA group, it is possible that the mechanisms responsible for this process are already maximally activated, potentially masking an effect of LA on lymph node colonisation.

### Fatty acid profiling of blood plasma

To examine the spectrum of fatty acids present in the duodenum (site of initial absorption), duodenal tissue sections were cut at necropsy from immunocompromised mice fed the various diets. Subsequent mass spectral analysis revealed an increase in duodenal LA concentrations in mice fed the VHLA diet (43.6±10.9 wt%) compared with mice fed the LLA diet (18.5±2.5 wt%) (Supplementary Table 3; *P*<0.02).

To determine circulating fatty acid levels, plasma was separated from whole blood collected at the time of necropsy. As in the duodenal fatty acid analysis, we observed an increase in plasma LA concentrations in mice of the VHLA group compared with mice of the LLA group (Supplementary Table 4). In contrast to duodenal tissue, plasma levels of AA were also found to be higher in mice fed the VHLA diet. These results confirmed that higher amounts of LA were absorbed in the small intestine, and that higher amounts of both LA and AA were present in circulating plasma of mice fed the VHLA diet.

### Stimulation of OCUM-2MD3 cell invasion through Matrigel by LA

**Figure 2:**
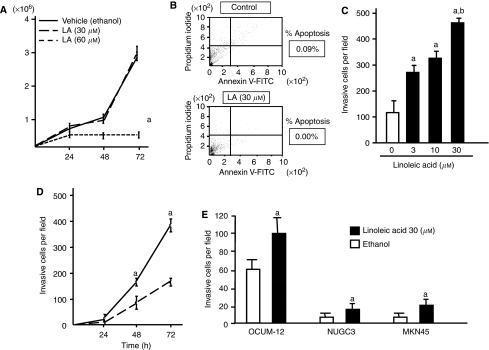
The effect of dietary LA on the invasion ability of OCUM-2MD3 cells through Matrigel. (**A**) Growth of OCUM-2MD3 cell lines after LA treatment. As described in the Materials and methods section, the number of viable OCUM-2MD3 cells with or without 30 *μ*M of LA treatment for the indicated time period was determined. (**B**) Apoptosis induction by LA. *Upper panel*: the typical examples of flowcytometic analysis in OCUM-2MD3 cells treated with vehicle (ethanol); apoptosis rate was 0.9%. *Lower panel*: analysis of cells treated with 30 *μ*M of LA; apoptosis rate was 0.0%. (**C**) Cells were treated for 45 min with vehicle (ethanol) or the indicated concentration of LA. Invasion across Matrigel-coated membranes was assessed after 72 h. a: Different from 0 *μ*M LA (*P*<0.01). b: Different from 3 and 10 *μ*M LA (*P*<0.05). (**D**) Cells were treated with ethanol (dotted line) or 30 *μ*M LA (solid line) and allowed to invade for the indicated time periods. a: Different from the ethanol-treated sample at the same time (*P*<0.05). (**E**) OCUM-12, NUGC3 and MKN-45 cells were treated with ethanol (dotted line) or 30 *μ*M LA (solid line). Invasion across Matrigel-coated membranes was assessed after 72 h. a: Different from 0 *μ*M LA (*P*<0.01). Values shown are mean±s.d. (*n*=4).

On the basis of the fact that the total tumour volumes in the VHLA group increased over those in either the LLA or HLA groups in mice, we investigated whether LA influenced the growth or apoptosis of OCUM-2MD3 cells by growth assay and flow cytometry. Contrary to prediction, LA treatment had no influence on the proliferation within 30 *μ*M, and showed cytotoxicity at 60 *μ*M (Figure 2A). Percentage of apoptosis in OCUM-2MD3 cells treated with 30 *μ*M LA was also not significantly altered (Figure 2B). Linoleic acid had no effect on adhesion of OCUM-2MD3 cells, as measured on Matrigel-coated membrane filters (data not shown). Therefore, we next investigated the effect of LA on the invasive capability of OCUM-2MD3 cells. Interestingly, LA strongly enhanced OCUM-2MD3 cell invasion through Matrigel (Figure 2C). The increased invasion occurred at both 48 and 72 h of incubation (Figure 2D). Similarly, 30 *μ*M AA, a metabolite of LA, increased the invasion of OCUM-2MD3 cells through Matrigel after 72 h (control=167±17 cells per field *vs* AA-treated=334±19 cells per field; *n*=3; *P*<0.01). Linoleic acid also increased invasion of other scirrhous gastric carcinoma cells, OCUM-12, NUGC3 and MKN-45, through Matrigel-coated membranes (Figure 2E).

### Role of ERK activity in the modulation of OCUM-2MD3 cell invasion

**Figure 3:**
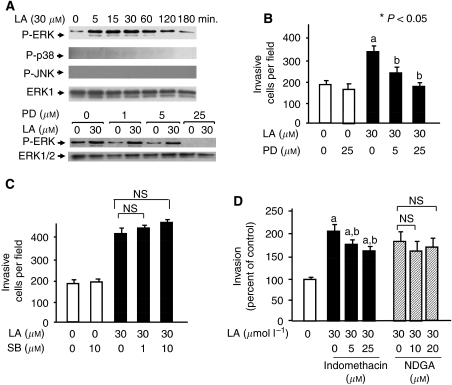
Linoleic acid induces activation of ERK MAPK, which is required for cell invasion. (**A**) *Upper panel*: linoleic acid (LA, 30 *μ*M) was added to suspended OCUM-2MD3 cells for the indicated time periods, after which lysates were analysed by SDS-polyacrylamide gel electrophoresis (50 *μ*g of protein per lane). *Lower panel*: cells were pre-treated with the ERK inhibitor, PD98059 (PD) at the indicated concentration for 60 min and treated with either 0 or 30 *μ*M LA for 30 min and then analysed as above. (**B**) OCUM-2MD3 cells were pre-treated with the ERK inhibitor, PD98059 (PD), and then with LA at the indicated concentrations and tested for invasion through Matrigel. a: Different from 0 *μ*M LA (*P*<0.05). b: Different from 30 *μ*M LA (*P*<0.05). (**C**) Cells were treated as above except that they were incubated in the presence or absence of the p38 inhibitor, SB203580 (SB), at the indicated concentrations, and tested for invasion. (**D**) Cells were treated as above except that they were incubated in the presence or absence of indomethacin or NDGA at the indicated concentration. a: Different from 0 *μ*M LA (*P*<0.05). b: Different from 30 *μ*M LA (*P*<0.05). Values shown are mean±s.d. (*n*=4). NS, not significant.

To examine possible mechanisms for how LA may stimulate invasion in these tumour cells, we examined whether signalling pathways involving MAPK (Palmantier *et al*, 1996, 2001; Paine *et al*, 2000) were involved in gastric carcinoma cell invasion modulated by dietary fatty acids. Serum-starved OCUM-2MD3 cells were exposed to 30 *μ*M LA for 5–180 min, and whole cell extracts were prepared. Linoleic acid treatment resulted in increased level of the phosphorylated form of ERK1/2, which was evident by 5 min and extended to at least 1 h (Figure 3A). In contrast, we observed no consistent phosphorylation of either p38 or SAPK/JNK after treatment with LA in OCUM-2MD3 cells (Figure 3A). PD98059, a specific inhibitor of ERK activation, efficiently blocked ERK phosphorylation (Figure 3A) and also blocked the LA-induced increase in OCUM-2MD3 cell invasion through Matrigel (Figure 3B). In contrast, the inhibitor did not block unstimulated cell invasion. SB203580, a specific p38 MAPK inhibitor, did not alter the LA-stimulated invasion ability of OCUM-2MD3 cells (Figure 3C). These results suggest that the ERK signalling pathway, but not p38 or JNK, is involved in LA-enhanced invasion by these human gastric carcinoma cells *in vitro*.

### A COX inhibitor blocks the invasion ability and metastasis of gastric carcinoma cells by LA

We next examined the effect of inhibitors of both LOX and COX on the ability of LA to stimulate invasion of the gastric carcinoma cells. Indomethacin, a general inhibitor of COX activity, decreased the LA-enhanced invasion of OCUM-2MD3 cells (Figure 3D). In contrast, the LOX inhibitor, NDGA, failed to inhibit invasion of this gastric carcinoma cell line through Matrigel. These data suggested that COX activity is required, in part, for LA-enhanced invasion *in vitro* and possibly LA-stimulated metastasis *in vivo*. We then examined the effects of indomethacin in the mouse model for experimental metastasis. As before, the incidence and size of visible tumour nodules in mice injected i.p. with OCUM-2MD3 cells were greater in the VHLA than in the LLA group (Table 3). Indomethacin had an inhibitory effect on both the number and volume of metastatic nodules per mouse in the LLA group. Similarly, the total volume of metastases per mouse was lower in the VHLA mice treated with indomethacin, compared with the control (ethanol) group. The incidence of tumour metastasis and the number of metastatic colonies were also reduced in the indomethacin-treated, VHLA group, although the reductions were not as large.

### COX-1, but not COX-2, is expressed in OCUM-2MD3 cells and in metastatic nodules

**Figure 4:**
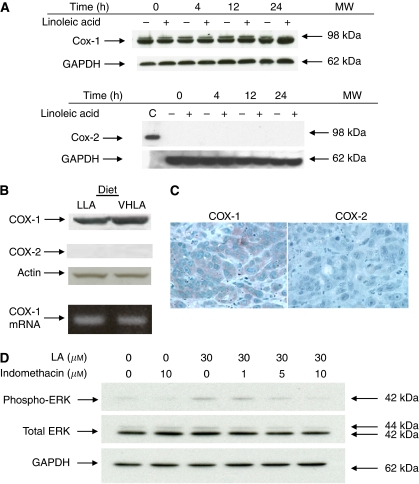
Cyclooxygenase expression in human OCUM-2MD3 cells and tumours. (**A**) Human gastric carcinoma cells were treated with vehicle or 30 *μ*M LA for the indicated time periods and then analysed by immunoblotting for COX-1 and COX-2. (**B**) Lysates were prepared from tumours dissected from nude mice and analysed either for protein by SDS-polyacrylamide gel electrophoresis (20 *μ*g protein per lane) or for mRNA by RT-PCR. (**C**) Expression of COX-1 and COX-2, as detected by immunohistochemistry. (**D**) OCUM-2MD3 cells were treated with vehicle (DMSO), 10 *μ*M indomethacin alone, 30 *μ*M LA alone or a combination of 30 *μ*M LA and varying concentrations of indomethacin. Total lysates (10 *μ*g protein per lane) were resolved by SDS-PAGE and immunoblotted for phospho-ERK, total ERK 1 and 2 and GAPDH.

The ability of indomethacin to block tumour cell metastasis and growth suggested that a COX was involved. Thus, we examined OCUM-2MD3 cells for the expression of the two isoforms of COX, COX-1 and COX-2. The COX-1 protein and mRNA were present in cultured OCUM-2MD3 cells, as determined by immunoblotting (Figure 4A) and RT-PCR (data not shown), respectively. We found no detectable levels of COX-2 protein in cultured cells (Figure 4A). After injection, metastatic nodules were obtained from both LLA- and VHLA-fed mice. Metastatic nodules taken from the two groups of mice expressed approximately equal levels of both COX-1 protein and COX-1 mRNA (Figure 4B). Immunohistochemistry also revealed COX-1 expression in metastatic nodules (Figure 4C). In contrast, we did not detect expression of COX-2 protein, either by immunoblotting or by immunohistochemical analysis (Figure 4B and C).

### Inhibition of COX activity prevents activation of ERK in OCUM-2MD3 cells

To determine whether COX activity is required for LA-induced phosphorylation of ERK in OCUM-2MD3 cells, the COX inhibitor indomethacin was used. Phosphorylation of ERK was inhibited by indomethacin (Figure 4C). These results support the hypothesis that COX activity is required for ERK activation in response to LA.

## Discussion

This study shows for the first time that scirrhous gastric carcinoma cells are impacted in their ability to metastasise in a mouse model by the dietary administration of (n-6)-PUFA. This is true both when tumour cells are injected into the i.p. space as well as when tumours are grafted onto the stomach wall. The levels of LA in the diets used (in terms of energy from LA) are higher than in most human diets (Arab, 2003). However, the relative abundance of LA measured in circulating mouse plasma in the VHLA group of our study are very similar to those measured in humans on certain high-fat diets (Skeaff *et al*, 2006), suggesting that the studies presented here are relevant to potential consequences in human disease. In this experimental metastasis study, the incidence of peritoneal metastasis, as well as the number of tumour nodules and total tumour volume, increased when LA was included at high levels in the diet. Although this model excludes the early steps of metastasis, gastric tumours are known to metastasise to the peritoneal cavity, and the presence of gastric tumour cells in the peritoneal cavity is a well-described marker for identifying patients who are at increased risk of peritoneal recurrence (Burke *et al*, 1998; Hayes *et al*, 1999). To better simulate the full metastatic cascade, we used an orthotopic graft model in which tumour sections, derived from tumours grown subcutaneously in a different mouse, were implanted on the stomach wall. This procedure led not only to efficient peritoneal metastasis but also to liver invasion and lymph node metastasis. Although the incidence of lymph node metastasis was not altered between LLA and HLA groups, liver invasion and peritoneal metastasis were higher in VHLA group. This result suggested that fatty acid intake may influence organ-specific metastasis of these cells, perhaps by altering the environment within host target sites and facilitating specific steps in the process of metastasis.

To address this question, we investigated the effect of LA on invasion, a critical step in the metastatic cascade. Linoleic acid increased OCUM-2MD3 cell invasion through Matrigel in an *in vitro* invasion assay. These results are consistent with reports indicating increased invasion of breast carcinoma cells treated with LA (Rose *et al*, 1994, 1995). Interestingly, LA did not affect the proliferation, apoptosis or adhesion of OCUM-2MD3 cells to Matrigel, indicating that increased invasion by LA was not caused by the enhancement of growth or adhesion of this cell line. To confirm that the response to LA is common in gastric carcinoma cells, we next sought to address whether other scirrhous gastric carcinoma cell lines had the similar response to LA. Interestingly, three cell lines, OCUM-12, NUGC3 and MKN-45, treated with LA showed a significant enhancement of invasive ability, indicating that LA effect might be common in scirrhous-type gastric cancer. These results contrast with previous reports in other types of tumour cells. For example, both our group and Johanning and Lin (1995) previously demonstrated that (n-6)-PUFA promoted adhesion of breast carcinoma cell lines to extracellular matrix. These differences may be the result of cell-specific signalling pathways. Nie *et al* (2000) described that eicosanoids regulated prostate cancer progression both positively and negatively, depending on the expression of enzymes such as COX, LOX and P450.

One mechanism by which fatty acids may enhance invasion is the secretion or activation of metalloproteases (Rose *et al*, 1994). We did not detect a change in the production or secretion of several metalloproteinases in OCUM-2MD3 cells stimulated with LA based on immunoblotting and gelatin zymography (data not shown). We also examined the expression of urinary plasminogen activator (UPA), an important factor known to induce proteolytic activity. Linoleic acid treatment did not induce UPA expression in OCUM-2MD3 cells, as measured by immunoblotting (data not shown).

We did find that the ERK inhibitor, PD98059, clearly reduced the LA-enhanced invasion of OCUM-2MD3 cells, suggesting that this signalling pathway has an important role in LA-stimulated gastric carcinoma cell invasion. Linoleic acid did not consistently activate p38 or SAPK/JNK, and the increased invasion was not sensitive to the p38 inhibitor SB203580, suggesting that these pathways are not necessary for LA-enhanced invasion in these cells. Other studies have shown that LA activates p38 and SAPK/JNK in stromal fibroblasts (Westermarck *et al*, 2000) and endothelial (Shin *et al*, 2001) cells. Therefore, it is likely that there is cell-type specificity with respect to LA activation of p38 and JNK pathways. Moreover, we found that pre-treatment of OCUM-2MD3 cells with indomethacin inhibited ERK phosphorylation *in vitro*, suggesting that the observed ERK activation is downstream of COX activity.

Our finding that a COX inhibitor, but not a LOX inhibitor, impaired LA-induced invasion of gastric carcinoma cells led us to investigate the effect of a COX inhibitor on peritoneal metastasis *in vivo*. Administration of indomethacin in drinking water inhibited the frequency and extent of peritoneal implantation, thus, serving as a proof of principle experiment for reducing tumour metastasis with inhibitors of fatty acid metabolism.

These results led us to examine the expression pattern of COX in OCUM-2MD3 cells. The COX-1, but not COX-2, was present in both cultured cells and metastatic nodules. Thus, it appears that COX-1 is primarily responsible for the enhanced metastatic effects of LA. As reviewed by Tsujii *et al* (1998), COX-1 expression regulates angiogenesis in endothelial cells. Therefore, it is conceivable that the ability of COX-1 to increase metastatic tumour formation *in vivo* may be through enhanced angiogenesis. Moreover, the unaltered expression of COX-1 in the presence of LA either in OCUM-2D3 cells or metastatic nodules *in vivo* suggests that any increase in LA metabolism may primarily be due to increased activity of COX-1, related to a higher substrate availability. Although our study has focused on COX expression in cultured cells and metastatic nodules, a complete understanding of the metastatic process will require measurement of the whole spectrum of LA metabolites, as a variety of biological effects, including dissemination and metastasis, may be exerted by several different eicosanoid metabolites (Mathias and Dupont, 1985).

A number of studies have suggested the importance of dietary fatty acids and their metabolism for human cancer development, either through animal models (Connolly *et al*, 1996), epidemiology of human populations (Armstrong and Doll, 1975) or cell culture systems (Horia and Watkins, 2007). Examples now exist in melanoma (Xia *et al*, 2006), hepatocellular (Rohr-Udilova *et al*, 2008), breast (Rose, 1997a), prostate (Angelucci *et al*, 2008) and pancreatic (Funahashi *et al*, 2008) cancers in which tumour cells are effected by (n-6)-PUFA. In some studies, (n-3)-PUFA appear to antagonise the effects of the (n-6) fatty acids (Funahashi *et al*, 2008), or have growth or metastasis inhibitory effects by themselves (Xia *et al*, 2006). Our work now adds gastric carcinoma to the list, and, when considered with the other models, suggests that specific components in the diet may influence a wide variety of cancers, making dietary modulation an attractive candidate for altering the aggressiveness of pre-existing cancers.

We (Palmantier *et al*, 1996; Nony *et al*, 2005) and others (Liu *et al*, 1991) have previously shown that metabolites of LA and AA can activate signalling pathways that regulate tumour cell behaviour. Our work with breast carcinoma cells showed that activation of TAK-1, a transforming growth factor receptor-associated kinase, is a key component of one of these pathways (Nony *et al*, 2005). It is interesting that others have now found the TGF-*β* pathway to be important for spreading and invasion of gastric carcinoma cells (Lee *et al*, 2005). This raises the question of whether metabolites of LA are working through the TGF-*β* pathway in gastric carcinoma cells.

Our results suggest that dietary LA promotes invasion and peritoneal metastasis of gastric carcinoma cells through an ERK-dependent pathway. Indomethacin treatment may contribute to impaired invasion and COX-1 activity. Results from our studies may provide information to suggest novel treatment and prevention options, as well as information useful in guiding the formulation of diets to enhance the protection of humans treated for cancer.

## Figures and Tables

**Table 1:** Effect of dietary fatty acid intake on incidence of grossly visible peritoneal metastatic nodules from OCUM-2MD3 human gastric carcinoma cells in nude mice

	**Dietary group**		
**Metastasis**	**LLA**	**HLA**	**VHLA**
Incidence	10/19 (53%)^a^	17/19 (89%)a,^b^	19/19 (100%)a,^b^
Total no.	28	43	79
<1 mm	1	2	6
1–3 mm	17	18	36
>3 mm	10	23	37
Nodules per mouse	1.5±2.0^c^	2.3±1.8^c^	4.2±2.7b,c,^d^
			
*Total tumour volume*			
Per mouse (mm^3^)	409±241^c^	657±991^c^	6674±9213b,c,^d^
Body weight	16.2±0.8^c^	17.1±1.2^c^	17.1±0.7^c^

Abbreviations: HLA=high linoleic acid; LLA=low linoleic acid; VHLA=very high linoleic acid.

a Values are means±s.e.m.

b Significantly different from LLA group; *P*<0.03.

c Values are means±s.e.m.

d Significantly different from HLA group; *P*<0.03.

**Table 2:** Effect of dietary fatty acid on growth of implanted gastric tumours and incidence of peritoneal metastasis nodules from OCUM-2MD3 cells in nude mouse

	**Dietary group**	
**Treatment group**	**LLA**	**VHLA**
*Gastric tumours*		
Incidence	9/9 (100%)	9/9 (100%)
Total volume (mm^3^)	5019±4971^a^	8512±5897^a^
		
*Metastasis*		
Peritoneum	1/9 (11%)	6/9 (67%)^b^
Lymph node	4/9 (44%)	6/9 (67%)
Liver invasion	2/9 (100%)	7/9 (78%)^b^
Body weight (g)	17.5±0.3^c^	16.5±0.7^c^

Abbreviations: LLA=low linoleic acid; VHLA=very high linoleic acid.

a Values are means±s.d.

b Significantly different from LLA group; *P*<0.03.

c Values are means±s.e.m.

**Table 3:** Effect of indomethacin on metastasis of OCUM-2MD3 cells in nude mice

	**LLA diet**		**VHLA diet**	
	**Ethanol**	**Indomethacin**	**Ethanol**	**Indomethacin**
Incidence	5/8 (63%)^a^	1/7 (14%)^a^	8/8 (100%)^a^	5/7 (71%)^a^
Total nodules	26	4^b^	46^b^	29c,^d^
Nodules per mouse	3.3±2.8^e^	0.6±1.5b,^e^	5.8±1.8c,^e^	4.1±3.2c,d,^e^
Total volume per mouse (mm^3^)	823.6±922.2^e^	25.1±66.5b,^e^	6155.9±4562.9c,^e^	2194.0±2408.6c,d,^e^
Body weight	16.7±0.8^a^	17.1±1.0^a^	17.3±0.9^a^	17.1±1.0^a^

Abbreviations: LLA=low linoleic acid; VHLA=very high linoleic acid.

a Values are means±s.e.m. (*n*=7 or 8).

b Significantly different from LLA ethanol;*P*<0.03.

c Significantly different from VHLA ethanol; *P*<0.03.

d Significantly different from LLA indomethacin; *P*<0.03.

e Values are means±s.d. (*n*=7 or 8).

## References

[bib1] Albini A, Iwamoto Y, Kleinman HK, Martin GR, Aaronson SA, Kozlowski JM, McEwan RN (1987) A rapid *in vitro* assay for quantitating the invasive potential of tumor cells. Cancer Res 47: 3239–3245 2438036

[bib2] Angelucci A, Garofalo S, Speca S, Bovadilla A, Gravina GL, Muzi P, Vicentini C, Bologna M (2008) Arachidonic acid modulates the crosstalk between prostate carcinoma and bone stromal cells. Endocr Relat Cancer 15: 91–100 18310278 10.1677/ERC-07-0100

[bib3] Arab L (2003) Biomarkers of fat and fatty acid intake. J Nutr 133(Suppl 3): 925S–932S 12612178 10.1093/jn/133.3.925S

[bib4] Armstrong B, Doll R (1975) Environmental factors and cancer incidence and mortality in different countries, with special reference to dietary practices. Int J Cancer 15: 617–631 1140864 10.1002/ijc.2910150411

[bib5] Braden LM, Carroll KK (1986) Dietary polyunsaturated fat in relation to mammary carcinogenesis in rats. Lipids 21: 285–288 3086654 10.1007/BF02536414

[bib6] Burke EC, Karpeh Jr MS, Conlon KC, Brennan MF (1998) Peritoneal lavage cytology in gastric cancer: an independent predictor of outcome. Ann Surg Oncol 5: 411–415 9718170 10.1007/BF02303859

[bib7] Connolly JM, Liu XH, Rose DP (1996) Dietary linoleic acid-stimulated human breast cancer cell growth and metastasis in nude mice and their suppression by indomethacin, a cyclooxygenase inhibitor. Nutr Cancer 25: 231–240 8771566 10.1080/01635589609514447

[bib8] De Vries CEE, van Noorden CJF (1992) Effects of dietary fatty acid composition on tumor growth and metastasis. Anticancer Res 12: 1513–1522 1444214

[bib9] Farrow DC, Vaughan TL, Hansten PD, Stanford JL, Risch HA, Gammon MD, Chow WH, Dubrow R, Ahsan H, Mayne ST, Schoenberg JB, West AB, Rotterdam H, Fraumeni Jr JF, Blot WJ (1998) Use of aspirin and other nonsteroidal anti-inflammatory drugs and risk of esophageal and gastric cancer. Cancer Epidemiol Biomarkers Prev 7: 97–102 9488582

[bib10] Fischer SM, Hagerman RA, Li-Stiles E, Lo HH, Maldve RE, Belury MA, Locniskar MF (1996) Arachidonate has protumor-promoting action that is inhibited by linoleate in mouse skin carcinogenesis. J Nutr 126: 1099S–1104S 8642440 10.1093/jn/126.suppl_4.1099S

[bib11] Folch J, Lees M, Stanley GHS (1957) A simple method for the isolation and purification of total lipids from animal tissues. J Biol Chem 226: 497–509 13428781

[bib12] Funahashi H, Satake M, Hasan S, Sawai H, Newman RA, Reber HA, Hines OJ, Eibl G (2008) Opposing effects of n-6 and n-3 polyunsaturated fatty acids on pancreatic cancer growth. Pancreas 36: 353–362 18437081 10.1097/MPA.0b013e31815ccc44

[bib13] Furstenberger G, Krieg P, Muller-Decker K, Habenicht AJR (2006) What are cyclooxygenases and lipoxygenases doing in the driver's seat of carcinogenesis? Int J Cancer 119: 2247–2254 16921484 10.1002/ijc.22153

[bib14] Hayes N, Wayman J, Wadehra V, Scott DJ, Raimes SA, Griffin SM (1999) Peritoneal cytology in the surgical evaluation of gastric carcinoma. Br J Cancer 779: 520–524 10.1038/sj.bjc.6690081 PMC2362407 10027323

[bib15] Horia E, Watkins BA (2007) Complementary actions of docosahexaenoic acid and genistein on COX-2, PGE2 and invasiveness in MDA-MB-231 breast cancer cells. Carcinogenesis 28: 809–815 17052999 10.1093/carcin/bgl183

[bib16] Hubbard NE, Erickson KL (1987) Enhancement of metastasis from a transplantable mouse mammary tumor by dietary linoleic acid. Cancer Res 47: 6171–6175 3677068

[bib17] Johanning GL, Lin TY (1995) Unsaturated fatty acid effects on human breast cancer cell adhesion. Nutr Cancer 24: 57–66 7491298 10.1080/01635589509514393

[bib18] Kujubu DA, Fletcher BS, Varnum BC, Lim RW, Herschman HR (1991) TIS10, a phorbol ester tumor promoter-inducible mRNA from Swiss 3T3 cells, encodes a novel prostaglandin synthase/cyclooxygenase homologue. J Biol Chem 266: 12866–12872 1712772

[bib19] Lala PK, Al-Mutter N, Orucevic A (1997) Effects of chronic indomethacin therapy on the development and progression of spontaneous mammary tumors in C3H/HEJ mice. Int J Cancer 73: 371–380 9359485 10.1002/(sici)1097-0215(19971104)73:3<371::aid-ijc12>3.0.co;2-g

[bib20] Lee MS, Kim TY, Kim YB, Lee LY, Lo SG, John HS, Kim TY, Bang YJ, Lee JW (2005) The signaling network of transforming growth factor beta 1, protein kinase C delta, and integrin underlies the spreading and invasiveness of gastric carcinoma cells. Mol Cell Biol 25: 6921–6936 16055706 10.1128/MCB.25.16.6921-6936.2005 PMC1190263

[bib21] Lichtenstein AH, Kennedy E, Barrier P, Danford D, Ernst ND, Grundy SM, Leveille GA, Van Horn L, Williams CL, Booth SL (1998) Dietary fat consumption and health. Nutr Rev 56: S3–19; discussion S19-28 10.1111/j.1753-4887.1998.tb01728.x 9624878

[bib22] Liu B, Timar J, Howlett J, Diglio CA, Honn KV (1991) Lipoxygenase metabolites of arachidonic and linoleic acids modulate the adhesion of tumor cells to endothelium via regulation of protein kinase C. Cell Regul 2: 1045–1055 1801923 10.1091/mbc.2.12.1045 PMC361904

[bib23] Lopez-Carrillo L, Lopez-Cervantes M, Ward MH, Bravo-Alvarado J, Ramirez-Espitia A (1999) Nutrient intake and gastric cancer in Mexico. Int J Cancer 83: 601–605 10521793 10.1002/(sici)1097-0215(19991126)83:5<601::aid-ijc5>3.0.co;2-6

[bib24] Mathias MM, Dupont J (1985) Quantitative relationships between dietary linoleate and prostaglandin (eicosanoid) biosynthesis. Lipids 20: 791–801 4068908 10.1007/BF02534404

[bib25] Matsuoka T, Hirakawa K, Chung YS, Yashiro M, Nishimura S, Sawada T, Saiki I, Sowa M (1998) Adhesion polypeptides are useful for the prevention of peritoneal dissemination of gastric cancer. Clin Exp Metastasis 16: 381–388 9626817 10.1023/a:1006573732238

[bib26] Michels KB, Mohllajee AR, Roset-Bahmanyar E, Beehler GP, Moysich KB (2007) Diet and breast cancer – A review of the prospective observational studies. Cancer 109: 2712–2749 17503428 10.1002/cncr.22654

[bib27] Nie D, Tang K, Szekeres K, Trikha M, Honn KV (2000) The role of eicosanoids in tumor growth and metastasis. Ernst Schering Res Found Workshop 31: 201–217 10.1007/978-3-662-04047-8_10 10943334

[bib28] Nishimura S, Chung YS, Yashiro M, Inoue T, Sowa M (1996) Role of alpha 2 beta 1- and alpha 3 beta 1-integrin in the peritoneal implantation of scirrhous gastric carcinoma. Br J Cancer 74: 1406–1412 8912536 10.1038/bjc.1996.556 PMC2074795

[bib29] Nony PA, Kennett SB, Glasgow WC, Olden K, Roberts JD (2005) 15(S)-Lipoxygenase-2 mediates arachidonic acid-stimulated adhesion of human breast carcinoma cells through the activation of TAK1, MKK6, and p38 MAPK. J Biol Chem 280: 31413–31419 16000313 10.1074/jbc.M500418200

[bib30] Paine E, Palmantier R, Akiyama SK, Olden K, Roberts JD (2000) Arachidonic acid activates mitogen-activated protein (MAP) kinase-activated protein kinase 2 and mediates adhesion of a human breast carcinoma cell line to collagen type IV through a p38 MAP kinase-dependent pathway. J Biol Chem 275: 11284–11290 10753939 10.1074/jbc.275.15.11284

[bib31] Palmantier R, George MD, Akiyama SK, Wolber FM, Olden K, Roberts JD (2001) Cis-polyunsaturated fatty acids stimulate beta1 integrin-mediated adhesion of human breast carcinoma cells to type IV collagen by activating protein kinases C-epsilon and -mu. Cancer Res 61: 2445–2452 11289113

[bib32] Palmantier R, Roberts JD, Glasgow WC, Eling TE, Olden K (1996) Regulation of the adhesion of a human breast carcinoma cell line to type IV collagen and vitronectin: roles for lipoxygenase and protein kinase C. Cancer Res 56: 2206–2212 8616873

[bib33] Park YH, Ryoo BY, Choi SJ, Kim HT (2004) A phase II study of capecitabine and docetaxel combination chemotherapy in patients with advanced gastric cancer. Br J Cancer 90: 1329–1333 15054450 10.1038/sj.bjc.6601724 PMC2409690

[bib34] Parkin DM, Pisani P, Ferlay J (1999) Global cancer statistics. CA Cancer J Clin 49: 33–64 10200776 10.3322/canjclin.49.1.33

[bib35] Prentice RL, Thomson CA, Caan B, Hubbell FA, Anderson GL, Beresford SAA, Pettinger M, Lane DS, Lessin L, Yasmeen S, Singh B, Khandekar J, Shikany JM, Satterfield S, Chlebowski RT (2007) Low-fat dietary pattern and cancer incidence in the women's health initiative dietary modification Randomized controlled trial. J Natl Cancer Inst 99: 1534–1543 17925539 10.1093/jnci/djm159 PMC2670850

[bib36] Rohr-Udilova NV, Stolze K, Sagmeister S, Nohl H, Schulte-Hermann R, Grasl-Kraupp B (2008) Lipid hydroperoxides from processed dietary oils enhance growth of hepatocarcinoma cells. Mol Nutr Food Res 52: 352–359 18293301 10.1002/mnfr.200700149

[bib37] Rose DP (1997a) Dietary fat, fatty acids and breast cancer. Breast Cancer 4: 7–16 11091570 10.1007/BF02967049

[bib38] Rose DP (1997b) Effects of dietary fatty acids on breast and prostate cancers: evidence from *in vitro* experiments and animal studies. Am J Clin Nutr 66: 1513S–1522S 9394709 10.1093/ajcn/66.6.1513S

[bib39] Rose DP, Connolly JM (1998) Influence of dietary linoleic acid on experimental human breast cancer cell metastasis in athymic nude mice. Int J Oncol 13: 1179–1183 9824628 10.3892/ijo.13.6.1179

[bib40] Rose DP, Connolly JM, Liu XH (1994) Effects of linoleic acid on the growth and metastasis of two human breast cancer cell lines in nude mice and the invasive capacity of these cell lines *in vitro*. Cancer Res 54: 6557–6562 7987856

[bib41] Rose DP, Connolly JM, Rayburn J, Coleman M (1995) Influence of diets containing eicosapentaenoic or docosahexaenoic acid on growth and metastasis of breast cancer cells in nude mice. J Natl Cancer Inst 87: 587–592 7752256 10.1093/jnci/87.8.587

[bib42] Rose DP, Hatala MA, Connolly JM, Rayburn J (1993) Effect of diets containing different levels of linoleic acid on human breast cancer growth and lung metastasis in nude mice. Cancer Res 53: 4686–4690 8402646

[bib43] Sauer LA, Blask DE, Dauchy RT (2007) Dietary factors and growth and metabolism in experimental tumors. J Nutr Biochem 18: 637–649 17418560 10.1016/j.jnutbio.2006.12.009

[bib44] Shin EY, Kim SY, Kim EG (2001) c-Jun N-terminal kinase is involved in motility of endothelial cell. Exp Mol Med 33: 276–283 11795492 10.1038/emm.2001.45

[bib45] Silva RA, Munoz SE, Guzman CA, Eynard AR, Evnard AR (1995) Effects of dietary n-3, n-6 and n-9 polyunsaturated fatty acids on benzo(a)pyrene-induced forestomach tumorigenesis in C57BL6J mice. Prostaglandins Leukot Essent Fatty Acids 53: 273–277 8577781 10.1016/0952-3278(95)90127-2

[bib46] Skeaff CM, Hodson L, McKenzie JE (2006) Dietary-induced changes in fatty acid composition of human plasma, platelet, and erythrocyte lipids follow a similar time course. J Nutr 136: 565–569 16484525 10.1093/jn/136.3.565

[bib47] Sowa M, Kato Y, Nishimura M, Yoshino H, Kubo T, Umeyama K (1989) Clinico-histochemical studies on type 4 carcinoma of the stomach--with special reference to mucopolysaccharides and sialic acid in tumor tissue. Jpn J Surg 19: 153–162 2470945 10.1007/BF02471579

[bib48] Tsujii M, Kawano S, Tsuji S, Sawaoka H, Hori M, DuBois RN (1998) Cyclooxygenase regulates angiogenesis induced by colon cancer cells. Cell 93: 705–716 9630216 10.1016/s0092-8674(00)81433-6

[bib49] Vanderhoek JY, Ekborg SL, Bailey JM (1984) Nonsteroidal anti-inflammatory drugs stimulate 15- lipoxygenase/leukotriene pathway in human polymorphonuclear leukocytes. J Allergy Clin Immunol 74: 412–417 6432882 10.1016/0091-6749(84)90140-4

[bib50] Westermarck J, Li S, Jaakkola P, Kallunki T, Grenman R, Kahari VM (2000) Activation of fibroblast collagenase-1 expression by tumor cells of squamous cell carcinomas is mediated by p38 mitogen-activated protein kinase and c-Jun NH2-terminal kinase-2. Cancer Res 60: 7156–7162 11156425

[bib51] Xia S, Lu Y, Wang J, He C, Hong S, Serhan CN, Kang JX (2006) Melanoma growth is reduced in fat-1 transgenic mice: impact of omega-6/omega-3 essential fatty acids. Proc Natl Acad Sci USA 103: 12499–12504 16888035 10.1073/pnas.0605394103 PMC1567907

[bib52] Yashiro M, Chung YS, Nishimura S, Inoue T, Sowa M (1996) Peritoneal metastatic model for human scirrhous gastric carcinoma in nude mice. Clin Exp Metastasis 14: 43–54 8521616 10.1007/BF00157685

[bib53] Zha S, Yegnasubramanian V, Nelson WG, Isaacs WB, De Marzo AM (2004) Cyclooxygenases in cancer: progress and perspective. Cancer Lett 215: 1–20 15374627 10.1016/j.canlet.2004.06.014

