# Supplementary material for: Elevated dietary linoleic acid increases gastric carcinoma cell invasion and metastasis in mice
Source: Br J Cancer. 2010 Sep 14;103(8):1182–91. doi: 10.1038/sj.bjc.6605881 (PMC2967057; doi:10.1038/sj.bjc.6605881)
Supplement: Supplementary Tables 1–4 [file 6605881x1.doc]

**Supplemental Table 1** Composition of Diets.

| **Constituent** | **LLA Diet, g/kg** | **HLA Diet, g/kg** | **VHLA Diet, g/kg** |
| --- | --- | --- | --- |
| Corn starch | 319.30 | 319.30 | 319.30 |
| Casein | 235.00 | 235.00 | 235.00 |
| Maltose dextrin | 70.00 | 70.00 | 70.00 |
| Dextrose | 13.00 | 13.00 | 13.00 |
| Cellulose | 59.00 | 59.00 | 59.00 |
| Mineral Mix^1^ | 41.10 | 41.10 | 41.10 |
| Vitamin Mix^2^ | 23.60 | 23.60 | 23.60 |
| Dl-Methionine | 3.50 | 3.50 | 3.50 |
| Choline bitartrate | 5.50 | 5.50 | 5.50 |
| Safflower oil | 24.20 | 101.00 | 178.30 |
| Coconut oil | 205.80 | 131.00 | 51.70 |

^1^ Supplying the following (g/kg of diet): carcium carbonate, 500.0; potassium citrate, 220.0; sodium chloride, 74.0; potassium sulfate, 52.0; magnesium oxide, 24.0; ferric citrate, 6.0; manganese carbonate, 3.5; zinc carbonate, 1.6; cupric carbonate, 0.3; potassium iodate, 0.01; chromium potassium sulfate, 0.55; sodium selinite, 0.01; sucrose, 118.03.

2 Containing (g/kg of diet): niacin, 3.00; calcium pantothenate, 1.60; pyridoxine HCl, 0.70; thiamine HCl, 0.60; riboflavin, 0.60; folic acid, 0.20; biotine, 0.02; Vitamin B_12_ (1%), 0.10; Vitamin E Acetate (50%), 20.00; Vitamin A Pantothenate (500k IU/g), 1.00; Vitamin D3 (400k IU/g), 0.25; menadione SB0.20; sacrose, 971.73.

**Supplemental Table 2 Fatty acid profiling of Diets.**

| Constituent | LLA Diet, g/100g | HLA Diet, g/100g | VHLA Diet, g/100g |
| --- | --- | --- | --- |
| **Fatty Acids Calculated as Triglycerides** |  |  |  |
| Saturated Fatty Acids (Acid Form) | 17.0 | 11.3 | 5.81 |
| Polysuturated Acids (Acid Form) | 1.54 | 6.08 | 9.28 |
| Omega 6 Fatty Acids | 1.6 | 6.35 | 9.69 |
| Total Fatty Acids | 20.1 | 20.2 | 18.9 |
| 9c 18:1 Oleic | 0.446 | 1.59 | 2.69 |
| 18:2 Linoleic | 1.6 | 6.35 | 9.68 |
| 20:0 Arachidic | 0.037 | 0.051 | 0.068 |
| 18:3 Gamma Linoleinic | <0.005 | <0.005 | <0.005 |
| 18:3 Linolenic | 0.008 | 0.014 | 0.019 |
| 20:4 Arachidonic | <0.005 | <0.005 | <0.005 |

**Supplemental Table 3** Percent of component fatty acids in the small intestine of LLA- and VHLA-fed mice.

|  | **LLA-fed Group**  **(n=4)** | **VHLA-fed Group**  **(n=4)** |
| --- | --- | --- |
| **Component Fatty Acid** | **Mean Wt % (± SD)** | **Mean Wt % (± SD)** |
| Lauric (12:0) | 19.30 ± 12.10 | 0.69 ± 0.47 ^a^ |
| Myristic (14:0) | 9.82 ± 6.61 | 0.38 ± 0.17 ^a^ |
| Palmitic (16:0) | 14.97 ± 1.62 | 13.28 ± 1.37 |
| Palmitoleic (16:1n-7) | 0.09 ± 0.03 | 0.02 ± 0.03 |
| Stearic (18:0) | 18.10 ± 3.79 | 16.22 ± 3.06 |
| Oleic (18:1n-9) | 2.53 ± 0.48 | 1.89 ± 0.64 |
| Linoleic (18:2n-6) | 18.52 ± 2.49 | 43.57 ± 10.94 ^a^ |
| γ Linolenic (18:3n-6) | 0.02 ± 0.02 | 0.00 ± 0.00 |
| Arachidic (20:0) | 0.04 ± 0.02 | 0.00 ± 0.01 |
| Gadoleic (20:1) | 0.06 ± 0.07 | 0.00 ± 0.00 |
| Dihomo γ Linolenic (20:3n-6) | 0.10 ± 0.08 | 0.00 ± 0.00 |
| Arachidonic (20:4n-6) | 9.30 ± 6.55 | 13.95 ± 4.49 |
| (20:3n-3) | 6.64 ± 4.71 | 9.74 ± 3.16 |
| Behenic (22:0) | 0.05 ± 0.11 | 0.00 ± 0.00 |
| Lignoceric (24:0) | 0.32 ± 0.23 | 0.05 ± 0.11 |
| Docosahexaenoic (22:6n-3) | 0.13 ± 0.08 | 0.24 ± 0.09 |

*^a^* Significantly different from LLA group, *P* < 0.05.

**Supplemental Table 4** Percent of component fatty acids in circulating blood plasma of LLA- and VHLA-fed mice.

|  | **LLA-fed Group**  **(n=4)** | **VHLA-fed Group**  **(n=4)** |
| --- | --- | --- |
| **Component Fatty Acid** | **Mean Wt % (± SD)** | **Mean Wt % (± SD)** |
| Myristic (14:0) | 2.30 ± 0.65 | 0.1 ± 0.14 ^a^ |
| Palmitic (16:0) | 17.5 ± 1.10 | 8.6 ± 3.69 ^a^ |
| Palmitoleic (16:1) | 1.2 ± 0.22 | 0.0 ± 0.00 ^a^ |
| Stearic (18:0) | 16.4 ± 0.60 | 13.6 ± 1.95 ^a^ |
| Oleic (18:1n-9) | 9.7 ± 0.87 | 2.9 ± 0.18 ^a^ |
| Linoleic (18:2n-6) | 27.1 ± 0.89 | 42.7 ± 12.47 ^a^ |
| γ Linolenic (18:3n-6) | 0.1 ± 0.03 | 0.2 ± 0.18 |
| α Linolenic (18:3n-3) | 0.0 ± 0.00 | 0.00 ± 0.00 |
| Arachidic (20:0) | 0.0 ± 0.00 | 0.0 ± 0.00 |
| Gadoleic (20:1) | 0.0 ± 0.00 | 0.00 ± 0.00 |
| Dihomo γ Linolenic (20:3n-6) | 0.7 ± 0.10 | 0.1 ± 0.15 |
| Arachidonic (20:4n-6) | 20.9 ± 2.51 | 29.5 ± 5.23 ^a^ |
| Behenic (22:0) | 0.0 ± 0.00 | 0.00 ± 0.00 |
| Brassic (22:2) | 3.2 ± 0.54 | 0.00 ± 0.00 ^a^ |
| Lignoceric (24:0) | 0.0 ± 0.00 | 0.00 ± 0.00 |
| Docosahexaenoic (22:6n-3) | 0.7 ± 0.10 | 0.9 ± 0.28 |

*^a^* Significantly different from LLA group, *P* < 0.05.
